# Impact of a two-phase robotic-assisted and home-based training program on falls and fall risk in older adults: a multicenter randomized controlled trial

**DOI:** 10.1186/s13063-025-09380-x

**Published:** 2025-12-20

**Authors:** Eunyoung Kwag, Alice De Luca, Giorgia Marchesi, Valentina Squeri, Philipp Ramm, Martin Hochheim, Max Wunderlich, Wiebren Zijlstra, Tobias Morat

**Affiliations:** 1 Institute of Movement and Sport Gerontology, German Sport University (GSU) Cologne, Am Sportpark Muengersdorf 6, Cologne, 50933 Germany; 2 Movendo Technology (MT), Via Bombrini 13/10, Genoa, 16149 Italy; 3 Generali Health Solutions GmbH (GHS), Hansaring 40-50, Cologne, 50670 Germany

## Abstract

**Background:**

Falls in older adults represent a significant health risk, and technology-supported interventions have emerged as a potential training solution to reduce fall risk. However, the effectiveness and applicability of such interventions require further evaluation. This study aimed to assess the impact of a novel technology-supported fall prevention program on fall incidence, fall risk, acceptability, and feasibility in community-dwelling older adults.

**Methods:**

In this multicenter randomized controlled trial, 292 older adults (aged ≥ 65 years) were randomly assigned at T_0_ (baseline) to either an intervention (INT) group or a control (CTR) group. The INT group completed 48 training sessions, commencing with the hunova robot and subsequently engaging in a home-based exercise program. The fall incidence from the initiation of training to the 1-year follow-up (T_3_) was monitored using fall diaries, while fall risk was evaluated through the administration of the Silver Index with the hunova robot and the timed-up-and-go test (TUG). The acceptability and feasibility of the intervention were evaluated via questionnaires, adherence, and dropout rates.

**Results:**

A total of 172 participants finished the study. After completing the intervention period and the 1-year follow-up, the CTR group demonstrated significant increases in both the total number of falls and the number of participants who experienced a fall (PEF) compared to baseline, whereas the INT group showed no significant changes. At follow-up, the incidence rate of falls did not differ significantly between groups, but the risk ratio of PEF was significantly higher in the CTR group after adjusting for baseline values. A significant time × group interaction was observed for the Silver Index, but not for the TUG. Adherence rates were high for both the technology-supported (83.6%) and home-based training (87.7%), with both phases well accepted by the participants.

**Conclusions:**

The two-phase technology-supported intervention demonstrated a potential for reducing falls and PEF, although there was no statistically significant difference between the two groups. The intervention was well received and demonstrated feasibility, indicating the potential for future implementation. Further research is required to investigate the cost-effectiveness of such programs, particularly in populations of older adults at elevated risk of falls.

**Trial registration:**

DRKS00025897. Registered on August 16, 2021.

**Supplementary Information:**

The online version contains supplementary material available at 10.1186/s13063-025-09380-x.

## Introduction

Falls are a leading, preventable cause of injury and loss of independence in older adults worldwide, with around one in three experiencing at least one fall annually [1, 2]. Beyond injuries and fear of falling, recurrent falls accelerate functional decline and healthcare utilization, underscoring the need for scalable, effective prevention strategies [3]. Increased postural sway is closely associated with a heightened risk of falls [4]. Furthermore, impaired physical functions, such as reduced gait speed, further elevate fall risk, regardless of cognitive status [5, 6]. Maintaining dynamic and reactive balance is critical for fall prevention, as fall rates tend to rise during activities that demand greater postural control [7]. Notably, dynamic balance is a key component of both usual and maximum gait speeds, which are important indicators of mobility [8]. Exercise remains the most consistently effective single-component strategy to reduce fall risk, particularly when balance and functional mobility are targeted [9], and multifaceted programs that integrate exercise, assistive technologies, and fall risk assessments offer additional benefits [10–12]. The typical duration of fall prevention exercise programs for older adults is approximately 25 weeks (see review by Ng et al. [13]). However, evidence suggests a dose-response relationship between fall rate reduction and program duration, with greater benefits observed in interventions lasting more than 32 weeks and/or performed two to five times per week [14–16]. Nevertheless, real-world implementation is limited by access, adherence, and sustainability challenges (e.g., transportation, supervision requirements, individualized progression) [12, 17, 18]. Technology-supported exercise programs, including sensor-based feedback, exergames, tele-rehabilitation, and robotic systems, have emerged to address these barriers by enabling objective assessment, adaptive training, and flexible delivery scale [19, 20]. Within this spectrum, robotic-assisted balance training and gait training, e.g., on a treadmill, can provide high-repetition, task-specific practice with standardized progression and real-time feedback, potentially enhancing training dose, motor learning, and safety for community-dwelling older adults [19, 20]. Evaluations suggest improvements in balance and mobility proxies; however, the evidence base is constrained by small samples, heterogeneous devices/protocols, short follow-up, and a predominant focus on surrogate outcomes rather than fall incidence [19–21]. In addition, reporting of acceptability (e.g., usability, user satisfaction) and feasibility (e.g., adherence, retention, adverse events, resource demands) is inconsistent, limiting judgments about broader implementation and cost-effectiveness [18, 22]. A pragmatic pathway to strengthen both effectiveness and sustainability is a sequential hybrid model: a supervised, center-based robotic-assisted phase to establish technique, safety, and adequate dose, followed by a tailored home-based program to support maintenance and real-world transfer [15, 16, 23, 24]. Although conceptually attractive, empirical evaluations of such hybrid, technology-supported programs with fall incidence as an outcome, alongside systematic reporting of acceptability and feasibility, remain scarce [19–21].

### Objective

We evaluated a two-phase fall prevention intervention comprising (a) a supervised robotic-assisted training and (b) a tailored home-based exercise component, as previously described in our study protocol [25]. The primary objective was to determine effects on fall incidence and fall risk in community-dwelling older adults. Secondary objectives were to assess acceptability (user satisfaction and experience) and feasibility (adherence, retention, adverse events) of the program. We hypothesized that the sequential technology-supported intervention would reduce falls and fall risk and would be acceptable and feasible in this population.

## Methods

This paper presents results from the multicenter randomized controlled trial “For more balance and muscle strength - Development, implementation and evaluation of a technology-based fall prevention program for community-dwelling older adults.” The trial was prospectively registered in the German Clinical Trial Register (registration number: DRKS00025897, registration date: 2021-08-16) and adhered to the revised Declaration of Helsinki and Germany’s data protection laws (i.e., General Data Protection Regulation of the German Data Protection Act). Ethical approval was obtained from the ethics committee of the German Sport University Cologne for all sites (Cologne, Bonn, Dusseldorf, Bremen), under application number 104/2021. For detailed study methodology and design, we refer to the published study protocol [25].

### Study design

This multicenter, randomized controlled longitudinal intervention trial included four assessment points: baseline (T_0_), post-technology-supported exercise (T_1_), post-home-based exercise (T_2_), and final follow-up after 12 months (T_3_) after T_2_. All measurements took place between 01/09/2021 and ended as planned on 13/12/2023. Measurements took place in Germany in one of the four study centers in Cologne, Bonn, Dusseldorf, or Bremen (see acknowledgements). Participants were randomly assigned as planned within the study protocol (see Morat et al. [25]) to the intervention group (INT) or the control group (CTR). The INT group completed a structured exercise program, while the CTR group continued their usual activities without intervention.

### Participants

Community-dwelling older adults insured by “Generali Deutschland Krankenversicherung AG” (Generali Germany Health Insurance AG) and its subsidiary insurance were recruited through personalized invitation letters from Generali Health Solutions GmbH. Eligibility criteria included:

- Age 65 years or older
- Residence within 20 km of a study center
- Increased fall risk (Silver Index score > 40%)
- Ability to walk more than 6 m unassisted

Exclusion criteria included medical conditions preventing participation and non-compliance with the hunova robot’s anthropometric requirements as detailed in the study protocol [25].

**Fig. 1:**
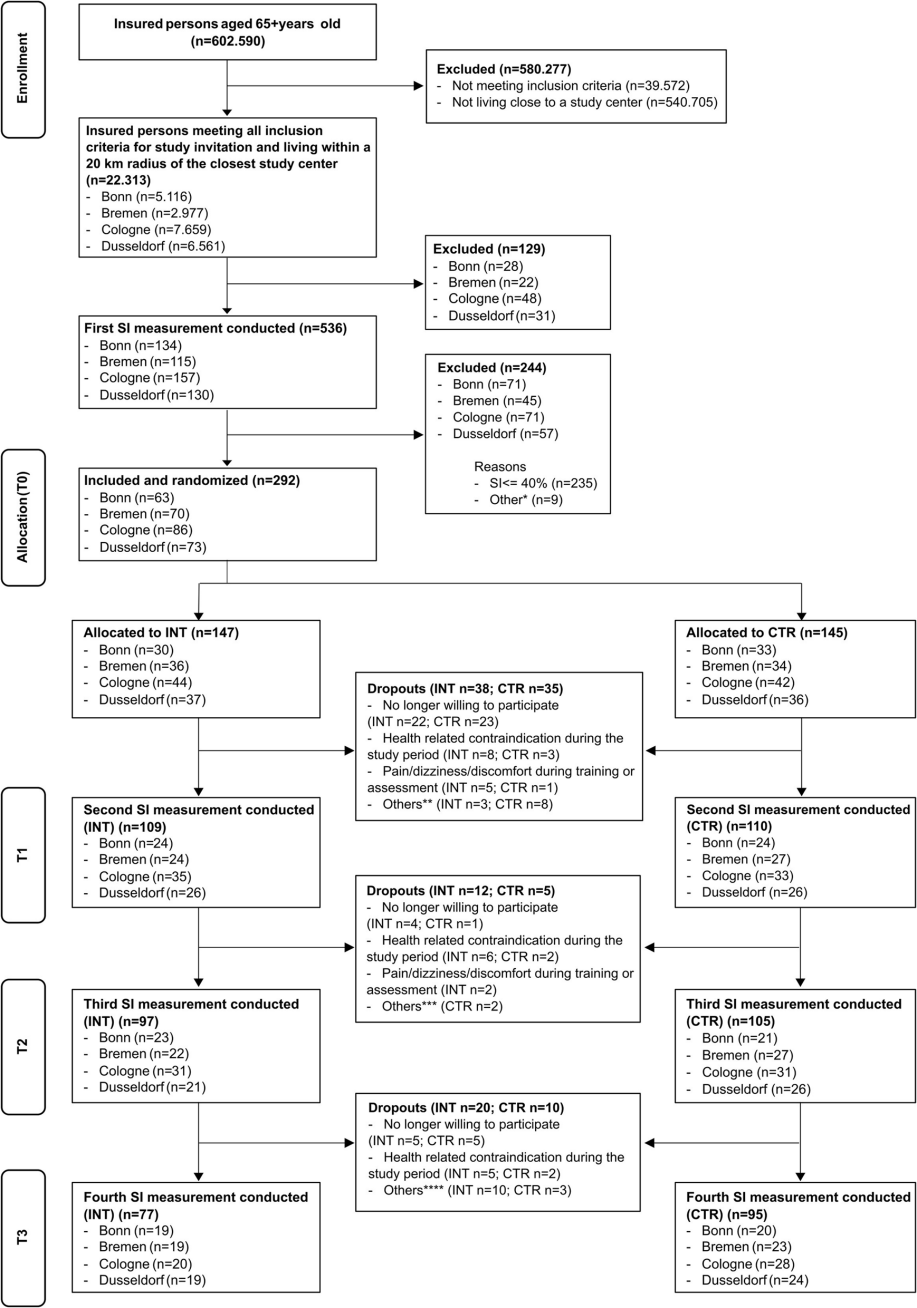
Flowchart of participants. Explanations for “Other” reasons: *Other exclusion reasons at T_0_ (*n* = 9): withdrew after the measurement (before group allocation) (*n* = 5); no Silver Index value available (*n* = 2); only wanted to participate in the initial measurement (*n* = 1); incorrectly excluded despite SI > 40% (*n* = 1), **Other dropout reasons until T_1_ (*n* = 11): inappropriate behavior towards the practice staff (*n* = 1); unwilling to participate in CTR (*n* = 2); did not agree with COVID-19 measures at the study center (*n* = 1); no reason given (*n* = 5); no longer reachable (*n* = 1); traveled (*n* = 1), ***Other dropout reasons until T_2_ (*n* = 2): traveled (*n* = 1); no longer reachable (*n* = 1), ****Other dropout reasons until T_3_ (*n* = 13): traveled (*n* = 1); no longer reachable (*n* = 2); unable to find a suitable appointment within the required time frame (*n* = 2); moved away (*n* = 2); no reason given (*n* = 6). The measurement (T_0_) of the first study participant took place on September 1, 2021. The T_3_ measurement of the last participant was completed on December 13, 2023. Note. CTR, control group; INT, intervention group; SI, Silver Index; T_0_, baseline measurement; T_1_, post-technology-supported exercise; T_2_, post-home-based exercise; T_3_, follow-up measurement

Informed consent was obtained from all participants prior to baseline assessment. The trial was powered for the annualized fall rate over 12 months (falls per person-year), analyzed using negative binomial regression with a log(person-time) offset to accommodate recurrent events and overdispersion. We specified an incidence rate ratio (IRR) for intervention versus control as the effect parameter. Based on the abridged Cochrane review of exercise for falls prevention in community-dwelling older adults, we considered a 23% relative reduction in fall rate (IRR = 0.77) as a realistic and clinically relevant target effect [26]. Based on an a priori sample size estimation using G*Power [27] and a negative-binomial framework (two-sided *α* = 0.05, 1:1 allocation, 12-month follow-up), the required total sample was 181 participants to achieve 80% power for detecting a 23% reduction in fall rate in a high-risk population. To align the calculation with our primary endpoint and a single primary study, we also derived parameters from a prior randomized controlled trial in at-risk older adults with 12-month falls follow-up [28]. Specifically, we used the control-arm fall rate and overdispersion reported from that study to parameterize a negative-binomial model for recurrent falls. Using the control fall rate and overdispersion from Barnett et al. [28] with the same *α*, power, allocation, and follow-up assumptions, the required total sample was *N* = 156. Given uncertainty in real-world control rates and dispersion across sites, we adopted the more conservative estimate (*N* = 181) to protect against under-powering. Allowing for 30% attrition (withdrawal, loss to follow-up, or <10 months of person-time), the target enrollment was *N* = 235. The flow of participants is shown in Fig. 1.

### Intervention (INT group)

#### Technology-supported exercise program (TSEP)

Upon recruitment and baseline assessment (T_0_), participants in the INT group began a 12–16-week technology-supported exercise program (TSEP) using the hunova robot (Movendo Technology, Genoa, Italy). The program consisted of 24 sessions (two per week), each lasting approximately 30 min. The hunova robot evaluates seven functional dimensions (static balance, dynamic balance, reactive balance, limits of stability, gait speed, sensory integration, sit to stand) to tailor exercises based on individual deficits. Electromechanical platforms offer multiple operational modes, and a display provides visual biofeedback, overseen by trained professionals.

#### Home-based exercise program (HBEP)

Following the TSEP, participants engaged in a home-based exercise program (HBEP) with a similar frequency and duration (24 sessions over 12–16 weeks). Initial sessions were supervised at the study centers, with subsequent home-based training guided by manuals and videos. The HBEP phase aimed to reinforce fall prevention strategies and improvements achieved during the TSEP. All detailed information regarding both training phases can be found in the study protocol [25].

### Outcome measures

General background data (date of birth, gender, height, weight, and a health questionnaire about current medications) was collected as part of the eligibility screening.

#### Fall diary

At T_0_, participants were asked to indicate the number of falls they had experienced over the past 12 months. Following this initial assessment, all participants were provided with fall diaries. From T_0_ onwards, participants recorded daily fall events in the diaries until the final measurement at T_3_. A fall was defined as “an unexpected event in which the participant comes to rest on the ground, floor, or lower level” [29]. In accordance with recommendations by Freiberger and Schoene [30], the fall diary distinguished between “nothing,” “tripping/stumbling,” “falling without injury,” and “falling with injury" (ranging from minor abrasions or contusions to more severe outcomes) [31]. The sum of the latter two categories (“falling without and with injury”) was taken as the total number of falls (in accordance with Lamb et al. [29]).

To avoid potential bias from using diaries covering less than a year, only fall diaries covering 12 months of the follow-up phase were used to evaluate the following measures: incidence rates (IR) and incidence rate ratios (IRR) of total falls, sum of falls without and with injury, as well as the proportion of participants who experienced a fall (≥1 fall during follow-up phase) (PEF) and risk ratios of PEF (RR) (for details see Statistical analysis).

#### Silver index

The Silver Index (SI), a composite fall risk score (0–100%) assessed by the hunova robot, incorporates balance tests and clinical factors (age, fall history, gait speed). A score above 40% was used as an inclusion criterion, assuming a higher risk of falls. The model underlying the multifactorial fall risk profile was developed and validated by Cella and colleagues [32]. SI was measured at T_0_, T_1_, T_2_, and T_3_.

#### Timed-up-and-go (TUG) test

The TUG test, a validated and reliable measure of mobility and fall risk [33], was administered at all four assessment points (T_0_, T_1_, T_2_, T_3_).

#### Questionnaires

At T_1_ and T_2_, participants in the INT group completed the following questionnaires to evaluate program acceptability:

- Task Load Index (TLX): Assessed subjective effort across six dimensions on a scale of 1–21 points per dimension (total 6–126 points), with higher scores (except in performance) indicating greater burden [34].
- Usefulness, Satisfaction and Ease of Use (USE) questionnaire: Measured user satisfaction across 30 items on a seven-point Likert scale (strongly disagree to strongly agree). The total score ranges from 30 to 210 points [35].
- User Experience Questionnaire (UEQ): Assessed program experiences on a seven-point Likert scale (−3 to 3) across six categories, with benchmarks (excellent, good, above average, below average, bad) for categorization [36].

Custom Feedback Questionnaire evaluated program enjoyment, motivation, intensity, duration, frequency, and likelihood of recommendation using Likert scales. While all questionnaires primarily evaluate acceptability, the custom feedback questionnaire, along with adherence (percentage of completed training sessions out of 24, percentage of participants in the INT group completing 100% of sessions, and percentage of participants in the INT group completing ≥75% of sessions for both TSEP and HBEP) and dropout rates, was used to assess the feasibility of the exercise program. Specifically, the training sessions for the HBEP were documented manually, whereas the training sessions for the TSEP were automatically recorded by the hunova robot.

#### Additional background information

Overall health status was assessed using the EQ-5D-5L questionnaire [37], and monthly physical activity was monitored by the German PAQ-50^+^ [38].

### Statistical analysis

Negative binomial regression and zero-inflated negative binomial regression were performed using Stata (version 18.5). All other data analysis was performed using IBM SPSS Statistics (version 29). Descriptive statistics were compared between INT and CTR groups using the Mann-Whitney U test for non-normally distributed or non-metric variables. Measurement differences across time points were evaluated using the Wilcoxon signed-rank test. Group differences in the descriptive results of falls and PEF were analyzed using the chi-square test, while time-based differences were assessed using the McNemar test and sign test.

The number of falls was reported as IR (falls per person-year, calculated as the total number of falls divided by the number of group participants), and the number of PEF as proportion (calculated as the total number of participants who experienced at least one fall divided by the total number of group participants), for each group at both T_0_ and T_3_, along with their respective 95% confidence intervals (CI). Group ratios for the IR and proportion were expressed as the IRR and RR, respectively. Group differences of fall incidence at T_3_ were analyzed using a negative binomial regression, both with and without adjustment of the number of falls at T_0_. Overdispersion was assessed prior to conducting the negative binomial regression; if overdispersion was not present, Poisson regression was used [39, 40]. Group differences in the proportion of PEF at T_3_ were analyzed using RR estimated via generalized linear models with a binomial distribution and log link function, both with and without adjustment for the number of PEF at T_0_.

A 2 × 4 repeated-measures ANOVA (group × time) was used to analyze SI and TUG data. Significant main or interaction effects were followed by Bonferroni post hoc tests. The significance level was set at *p* < 0.05.

## Results

The randomization process was effective; at T_0_, there were no significant differences between the INT and CTR groups (see Appendix for baseline characteristics of all participants at T_0_, who could be included based on the defined inclusion and exclusion criteria, *n* = 292). A total of 172 participants (59% of the initial cohort) completed the follow-up at T_3_ and were included in the final analysis of primary and secondary outcomes. Baseline characteristics of these participants are summarized in Table 1.

**Table 1:** Baseline characteristics of the study participants included at T_3_

	INT (*n* = 77)	CTR (*n* = 95)	*p*
Gender, female (*n* (%))	30 (39)	38 (40)	0.890
Age (years)	72 (6)	74 (5)	*0.008*
Body height (cm)	174 (9)	173 (9)	0.957
Body mass (kg)	84 (15)	82 (15)	0.988
BMI (kg/m^2^)	28 (4)	27 (4)	0.357
SI value at T_0_ (%)	66.1 (17.1)	62.5 (16.5)	0.188
TUG time at T_0_ (s)^*^	8.3 (1.9)	8.1 (1.6)	0.741
Gait speed at T_0_ (m/s)	1.2 (0.2)	1.3 (0.2)	0.144
Falls in the past year at T_0_ (*n* (%))			0.191^a^
No falls	47 (61.0)	67 (70.5)	
Fall(s)	30 (39.0)	28 (29.5)	
Overall health status at T_0_ (EQ-5D-5L)^*^			
Mobility	1.3 (0.6)	1.1 (0.3)	0.232
Self-care	1.0 (0.1)	1.1 (0.4)	0.197
Usual activities	1.1 (0.3)	1.1 (0.4)	0.208
Pain/discomfort	1.5 (0.6)	1.4 (0.6)	0.806
Anxiety/depression	1.1 (0.5)	1.1 (0.3)	0.981
Health status	79.8 (14.0)	82.3 (13.0)	0.274
Physical activity at T_0_ (German PAQ-50^+^)^†^			
Activity (MET-hours/week)	134.7 (69.0)	145.4 (88.2)	0.845
Energy (kcal/week)	10,602.0 (5721.0)	11,498.3 (7238.4)	0.910

Data are given as mean (*SD* Standard deviation) unless stated otherwise

*INT* Intervention group, *CTR* Control group, *BMI* Body Mass Index, *SI* Silver Index, *T*_*3*_ Follow-up measurement, *TUG* Timed-up-and-go test

^a^Detailed descriptive results of fall data, evaluated using fall diaries, are provided in Tables 2 and 3

^*^n: INT= 75, CTR: 93

^†^*n*: INT = 62, CTR: 83

Except for age, there were no significant group differences in baseline data of participants who completed the follow-up. However, the CTR group in the final analysis had experienced fewer falls compared to the initial CTR group (at T_0_) as well as compared to the INT group at T_0_ and T_3_ (see Table 1, and see Appendix).

**Table 2:** Descriptive results of the number of falls

a								
Group		*M*	*SD*	Min	Max	Percentiles		
						25.	50. (Median)	75.
INT	T_0_	0.7	1.1	0	5	0	0	1
	T_3_	0.8	2.2	0	13	0	0	1
CTR	T_0_	0.5	1.0	0	6	0	0	1
	T_3_	0.8	1.4	0	10	0	0	1
b								
*n* (%)	Total	T_0_			T_3_			
Category		0	1	2	0	1		2
INT	67	38 (57)	17 (25)	12 (18)	47 (70)	8 (12)		12 (18)
CTR	90	66 (73)	14 (16)	10 (11)	49 (54)	29 (32)		12 (13)
Total	157	104	31	22	96	37		24

a represents the total number of falls per person, while b shows the categorical distribution at the baseline (T0) and follow-up (T3) measurements

*M* mean, *SD* standard deviation, *Min* minimum, *Max* maximum, *INT* intervention group, *CTR* control group, *T*_*0*_ baseline measurement, *T*_*3*_ follow-up measurement; category 0: no fall, 1: one fall, 2: two or more falls

**Table 3:** Descriptive results of the number of participants who experienced a fall (PEF)

a					
*n* (%)	Total	T_0_		T_3_	
Category		No	Yes	No	Yes
INT	67	38 (57)	29 (43)	47 (70)	20 (30)
CTR	90	66 (73)	24 (27)	49 (54)	41 (46)
Total	157	104	53	96	61
b					
Group			T_3_		
			No	Yes	Total
INT	T_0_	No	31	7	38
		Yes	16	13	29
		Total	47	20	67
CTR		No	37	29	66
		Yes	12	12	24
		Total	49	41	90

a represents the total number of participants who experienced a fall (PEF) at the baseline (T_0_) and follow-up (T_3_) measurements. b is a cross table indicating the ratio of the number of PEF

*INT* intervention group, *CTR* control group, *T0* baseline measurement, *T3* follow-up measurement

Sample sizes for specific outcomes differed due to missing data: SI (*n* = 172; INT = 77, CTR = 95), TUG (*n* = 159 after outlier removal; INT = 70, CTR = 89), adherence (INT, TSEP (T_0_ toT_1_) *n* = 109, HBEP (T_1_ to T_2_) *n* = 85), and fall diary (*n* = 157; INT = 67, CTR = 90).

### Primary outcomes

#### Falls and participants who experienced a fall (PEF)

At baseline (T_0_), although the INT group had a higher number of falls, the difference in the number between the INT and CTR groups was not statistically significant (*χ*^2^(2) = 4.743, *p* = 0.093, Table 2). However, the distribution of falls at T_3_ differed significantly between the groups (*χ*^2^(2) = 8.780, *p* = 0.012). The INT group showed no significant changes in the number of falls from T_0_ to T_3_ (*z* = −1.278, *p* = 0.201). In contrast, the CTR group demonstrated a significant increase during the same period (*z* = −2.087, *p* = 0.037). Due to low cell frequencies at T_3_, a chi-square test for the number of falls with injury could not be performed.

The number of individuals who experienced one or more falls (PEF) is summarized in Table 3. The number of PEF differed significantly between the groups at both T_0_ (*χ*^2^(1) = 4.743, *p* = 0.029) and T_3_ (*χ*^2^(1) = 3.987, *p* = 0.046). While the decrease in the number of PEF from T_0_ to T_3_ in the INT group was not statistically significant (*p* = 0.093), a significant increase was observed in the CTR group (*χ*^2^(1, *n* = 90) = 6.24, *p* = 0.012). In addition, 17% of all participants across both groups who experienced falls reported falls with injury. The number of PEF with injuries in the INT group significantly decreased from T_0_ to T_3_ (*p* = 0.002), while no significant change was observed in the CTR group (*χ*^2^(1, *n* = 90) = 2.70, *p* = 0.100). Furthermore, no significant difference between the groups was observed at T_3_ (*χ*^2^(1) = 0.399, *p* = 0.527).

#### Incidence rate of falls and proportion of participants who experienced a fall (PEF)

At T_0_, IR of falls per person-year was significantly higher than zero in both groups (INT: 0.746, 95% CI: 0.449 to 1.043, *p* < 0.001; CTR: 0.456, 95% CI: 0.277 to 0.635, *p* < 0.001). However, there was no significant difference in the IR between the groups (IRR = 1.638, 95% CI: 0.936 to 2.866, *p* = 0.084).

Also, at T_3_, the IR of falls per person-year was significantly greater than zero in both groups: INT = 0.836 (95% CI: 0.469 to 1.203, *p* < 0.001) and CTR = 0.778 (95% CI: 0.479 to 1.076, *p* < 0.001) (see Table 4). Negative binomial regression models indicated that the INT group had a 10.3% lower IR compared to the CTR group after adjusting for the number of falls at T_0_, whereas without this adjustment, the INT group had a 7.5% higher IR compared to the CTR group. However, neither model showed a statistically significant difference in the IR between the groups (see Table 4a and b). The negative binomial regression, adjusted for the number of falls at T_0_, showed that the number of falls at T_0_ significantly influenced the post-intervention fall rate (*p* = 0.003). Both models met the assumption of overdispersion for conducting negative binomial regression (*p* < 0.001). In addition, the IR of falls with injury was significantly greater than zero in both groups at T_3_: INT = 0.299 (95% CI: 0.117 to 0.480, *p* = 0.001) and CTR = 0.222 (95% CI: 0.096 to 0.349, *p* = 0.001). However, there was no significant difference in the IR of falls with injury between the INT and CTR groups (IRR without adjustment for the number of falls at T_0_ = 1.343, 95% CI: 0.584 to 3.088, *p* = 0.487; IRR with adjustment = 1.149, 95% CI: 0.504 to 2.619, *p* = 0.741).

**Table 4:** Incidence rate of falls and proportion of participants who experienced a fall (PEF) with group comparisons at T_3_

		Number of falls			Number of PEF		
		IR	95% CI	*p*^§^	Proportion	95% CI	
	INT	0.836	0.469–1.203	< 0.001	0.299	0.201–0.419	
	CTR	0.778	0.479–1.076	< 0.001	0.456	0.355–0.560	
a		IRR	95% CI	*p*	RR	95% CI	*p*
	Group	1.075	0.560–1.925	0.809	0.655	0.426–1.008	0.055
		*α*		*p*^#^			
		2.162	1.362–3.434	< 0.001			
b		IRR	95% CI	*p*	RR	95% CI	*p*
	Group	0.897	0.503–1.601	0.714	0.640	0.418–0.980	0.040
	Falls/PEF at T_0_	1.516	1.148–2.003	0.003	1.406	0.957–2.065	0.083
		*α*		*p*^#^			
		1.825		<0.001			

This represents the number of falls and participants who experienced a fall (PEF) per year at the follow-up measurement for both groups (i.e., incidence rate (IR) and proportion). The incidence rate ratio (IRR: IR of the INT group/IR of the CTR group) of the number of falls was analyzed using a zero-inflated negative binomial regression, both unadjusted (a) and adjusted for baseline falls or PEF (b). The risk ratio (RR: Proportion of the INT group/Proportion of the CTR group) of the number of PEF was analyzed using a generalized linear model

*CI* Confidence interval, *CTR* Control group, *INT* Intervention group, *T*_*0*_ Baseline measurement, *T*_*3*_ Follow-up measurement

*α*: The alpha parameter measures the degree of overdispersion. If alpha > 0, overdispersion meets

^§^The *z*-statistic is used to test whether the incidence rate of each group is significantly different from zero

^#^LR test of alpha = 0 is used to test for overdispersion in the data when fitting a negative binomial model

At T_0_, the proportion of PEF was higher in the INT group compared to the CTR group (INT: 0.433, 95% CI: 0.319 to 0.554; CTR: 0.267, 95% CI: 0.185 to 0.368) with a significant difference between groups (RR = 1.623, 95% CI: 1.047 to 2.517, *p* = 0.030).

The proportion of PEF at T_3_ was 0.299 (95% CI: 0.201 to 0.419) in the INT group and 0.456 (95% CI: 0.355 to 0.560) in the CTR group, with no statistically significant difference observed between the groups (RR = 0.655, 95% CI: 0.426 to 1.008, *p* = 0.055) (Table 4). However, the model adjusted for the number of PEF at T_0_ indicated that the proportion of PEF in the INT group was significantly lower (−36%) than that in the CTR group at T_3_ (RR = 0.640, 95% CI: 0.418 to 0.9580, *p* = 0.040), with the number of PEF at T_0_ showing a not significant influence on the proportion of PEF (*p* = 0.083). In addition, the proportion of PEF with injury did not differ significantly between the groups at T_3_, either without adjustment (RR = 1.247, 95% CI: 0.628 to 2.476, *p* = 0.527) or with adjustment for the number of PEF at T_0_ (RR = 1.160, 95% CI: 0.582 to 2.311, *p* = 0.674).

### Secondary outcomes

#### Fall risk

**Fig. 2:**
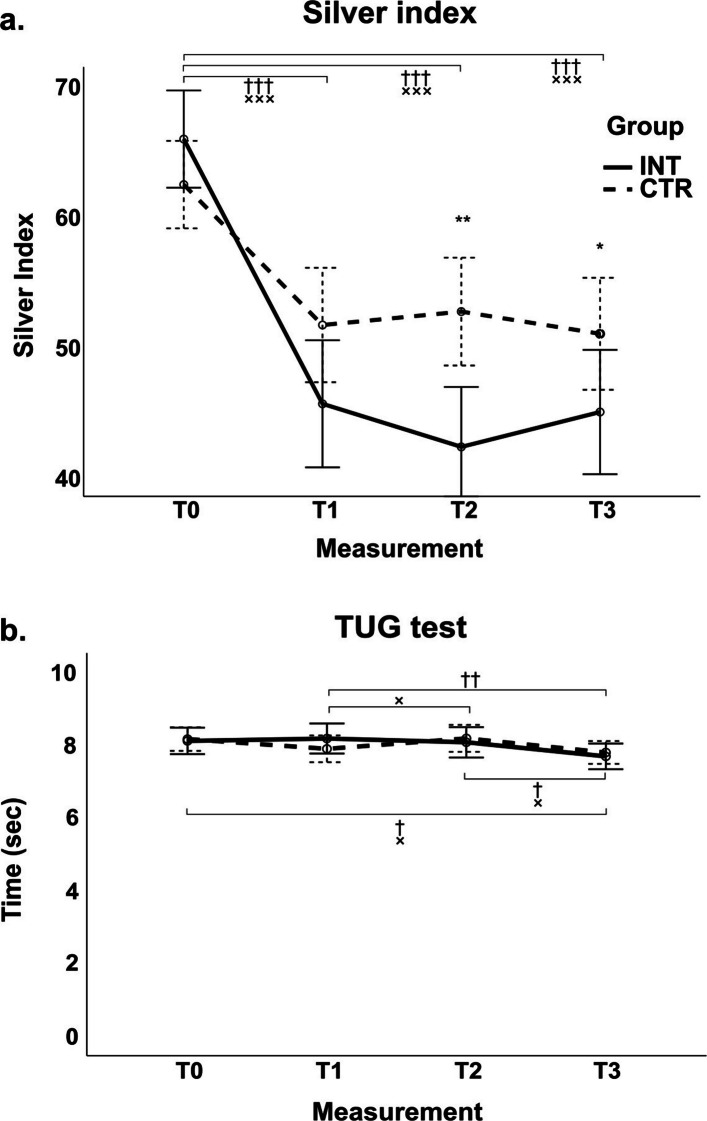
Fall risk assessed by Silver Index and TUG test across four measurements. Intervention group (INT) is represented by a solid line, whereas control group (CTR) by a dashed line. Differences between measurements are indicated by † for the INT group and × for the CTR group. Differences between groups are denoted by an asterisk (*). If the *p* value is less than 0.05, it is indicated by, e.g., †; if *p* < 0.01, by ††; if *p* < 0.001, by †††. Note. CTR, control group; INT, intervention group; T_0_, baseline measurement; T_1_, post-technology-supported exercise; T_2_, post-home-based exercise; T_3_, follow-up measurement; TUG, timed-up-and-go test

Fall risk was assessed using the SI and TUG across all time points (T_0_, T_1_, T_2_, T_3_; Table 5). For the SI, there was a significant main effect of time (*F* = 42.902, *p* < 0.001, partial *η*^2^ = 0.202), a significant main effect of group (*F* = 5.528, *p* = 0.020, partial *η*^2^ = 0.031), and a significant time × group interaction (*F* = 5.716, *p* < 0.001, partial *η*^2^ = 0.033). Post hoc results are indicated in Fig. 2a. For the TUG, a significant main effect of time was found (*F* = 5.148, *p* = 0.002, partial *η*^2^ = 0.032). However, there was no significant main effect of group (*F* = 0, *p* = 0.983, partial *η*^2^ < 0.001) and no time × group interaction (*F* = 1.317, *p* = 0.268, partial *η*^2^ = 0.008; see Fig. 2b).

**Table 5:** Fall risk assessed by Silver Index and TUG test across four measurements in intervention and control groups

Measurement	Group	Silver Index	TUG
		*M* (± *SD*)	*M* (± *SD*)
T_0_	INT	66.1 (17.1)	8.1 (1.5)
	CTR	62.5 (16.5)	8.2 (1.6)
	Total	64.1 (16.8)	8.2 (1.5)
T_1_	INT	45.5 (21.9)	8.2 (2.0)
	CTR	51.6 (21.9)	7.9 (1.6)
	Total	48.9 (22.1)	8.0 (1.8)
T_2_	INT	42.2 (21.4)	8.1 (1.9)
	CTR	52.7 (20.0)	8.2 (1.7)
	Total	48.0 (21.3)	8.2 (1.8)
T_3_	INT	43.6 (20.9)	7.7 (1.6)
	CTR	51.0 (20.8)	7.8 (1.5)
	Total	48.2 (21.6)	7.8 (1.5)

*M* mean, *SD* standard deviation, *INT* intervention group, *CTR* control group, *T*_*0*_ baseline measurement, *T*_*1*_ post-technology-supported exercise, *T*_*2*_ post-home-based exercise, *T*_*3*_ follow-up measurement, *TUG* timed-up-and-go test

#### Acceptability

Descriptive statistics for acceptability measures are presented in Table 6. The TLX indicated that physical demand (*Z* = −2.742, *p* = 0.006) and effort (*Z* = −2.097, *p* = 0.036) were significantly higher during the TSEP compared to the HBEP. Temporal demand was higher in the HBEP (*Z* = −3.620, *p* < 0.001). No significant differences were found for mental demand, performance, or frustration.

**Table 6:** Acceptability and feasibility after each TSEP and HBEP

Measurement time		T_1_	T_2_	*Z*	*p*
Questionnaire		*M* (*SD*)	*M* (*SD*)		
TLX (*n* = 108)	Mental demand	7.31 (5.07)	6.72 (4.95)	−1.611	0.107
	Physical demand	10.76 (4.80)	9.20 (3.89)	−2.742	*0.006*
	Temporal demand	6.40 (4.43)	8.41 (5.10)	−3.620	<*0.001*
	Performance	8.56 (5.63)	8.17 (5.69)	−1.051	0.293
	Effort	9.14 (5.20)	7.99 (4.45)	−2.097	*0.036*
	Frustration	5.35 (4.69)	4.88 (4.35)	−0.686	0.493
	Total	47.53 (19.20)	45.37 (20.86)	−1.533	0.125
USE (*n* = 107)	Usefulness	38.19 (10.60)	35.01 (11.27)	−2.696	*0.007*
	Ease of use	59.19 (11.23)	57.89 (12.84)	−0.817	0.414
	Ease of learning	22.51 (5.21)	23.04 (5.24)	−0.698	0.485
	Satisfaction	37.52 (8.86)	35.81 (9.94)	−1.974	*0.048*
	Total	157.41 (30.76)	151.76 (32.95)	−1.965	*0.049*
UEQ (*n* = 108)	Attractiveness	1.78 (0.87)	1.35 (0.92)	−3.615	<*0.001*
	Perspicuity	1.69 (0.87)	1.63 (0.99)	−0.654	0.513
	Efficiency	1.07 (0.81)	0.92 (0.76)	−2.504	*0.012*
	Dependability	1.25 (0.83)	1.46 (0.81)	−1.971	*0.049*
	Stimulation	1.54 (1.02)	0.95 (1.06)	−4.828	<*0.001*
	Novelty	1.20 (0.99)	0.33 (0.96)	−5.946	<*0.001*
General feedback* (*n* = 108)	Fun	5.61 (0.58)	4.96 (1.02)	−5.565	<*0.001*
	Motivation	5.50 (0.78)	4.60 (1.08)	−6.286	<*0.001*
	Intensity	3.46 (0.94)	2.96 (0.68)	−4.267	<*0.001*
	Duration	2.98 (0.67)	3.09 (0.49)	−0.852	0.394
	Frequency	2.90 (0.43)	3.02 (0.58)	−1.171	0.242
	Recommendation	8.90 (2.40)	7.85 (3.05)	−2.959	*0.003*

*Likert scales were varied: “Fun” was rated on a six-point scale (strongly disagree to strongly agree); “Motivation” on a six-point scale (very low to very high); “Frequency” from 1 (too infrequent), 3 (just right), to 5 (too frequent); and “Recommendation” on an 11-point scale (very unlikely to very likely)

*M *mean, *SD* standard deviation, *INT* intervention group, *CTR* control group, *T*_*1*_ post-technology-supported exercise (TSEP), *T*_*2*_ post-home-based exercise (HBEP), *TLX* Task Load Index, *USE* Usefulness, Satisfaction and Ease of Use questionnaire, *UEQ* User Experience Questionnaire

The USE showed higher total scores for the TSEP compared to the HBEP (*Z* = −1.965, *p* = 0.049), with the TSEP receiving significantly higher scores for usefulness (*Z* = −2.696, *p* = 0.007) and satisfaction (*Z* = −1.974, *p* = 0.048). No significant differences were found for ease of use or ease of learning between the two training phases, TSEP and HBEP.

In the UEQ, the TSEP was rated significantly higher than the HBEP in attractiveness (*Z* = −3.615, *p* < 0.001), efficiency (*Z* = −2.504, *p* = 0.012), stimulation (*Z* = −4.828, *p* < 0.001), and novelty (*Z* = −5.946, *p* < 0.001). Conversely, dependability was rated higher for the HBEP (*Z* = −1.971, *p* = 0.049), while no significant difference was found for perspicuity.

#### Feasibility

General feedback indicated that participants rated TSEP higher than HBEP for fun (*Z* = −5.565, *p* < 0.001), motivation (*Z* = −6.286, *p* < 0.001), intensity (*Z* = 4.267, *p* < 0.001), and likelihood of recommendation (*Z* = −2.959, *p* = 0.003). No significant differences were observed for duration, frequency, positive effects, or pain. The mean adherence rate during the TSEP training phase was 83.6%, with 40 participants (37%) completing all 24 training sessions and 83 participants (76%) completing more than 75% of the 24 training sessions. For HBEP, mean adherence was 87.7%, with 62 participants (73%) completing all 24 training sessions and 76 participants (89%) completing more than 75% of the 24 training sessions. On average, the training period lasted 13.6 weeks for TSEP and 13.1 weeks for HBEP.

The dropout rate was 26% for TSEP and 11% for HBEP. The overall dropout rate for the study was 41%, with 172 participants completing the T_3_ measurement, slightly below the projected target of 181 participants [25].

## Discussion

This study aimed to evaluate whether a two-phase exercise program incorporating robot-assisted and home-based training could reduce fall incidence (primary outcome) and fall risk, as measured by the Silver Index (SI) and timed-up-and-go test (TUG) (secondary outcomes). As additional secondary outcomes, the program’s acceptability and feasibility were assessed. The number of falls and PEF in the intervention (INT) group in our study was not significantly reduced in comparison to the control (CTR) group following the completion of the exercise program. However, the INT group demonstrated a positive trend with a 10.3% lower incidence rate (IR) of falls and a significantly lower proportion of PEF (−36%) at follow-up compared to the CTR group, after adjusting for baseline incidence. Fall risk, assessed by the SI, significantly improved in the INT group at follow-up, while TUG scores did not show a clinically relevant difference between groups. Both training programs appeared to be reasonably acceptable and feasible, with high adherence and a modest dropout rate among participants.

### Fall incidence and fall experience following the two-phase exercise program

Systematic reviews have identified individually tailored and multifactorial exercise interventions as effective fall prevention programs for older adults [41–43]. For preventing falls, a tailored training program followed by a home-based exercise program (HBEP) appears to be a cost-effective approach in older adults [42]. However, the impact of fall-preventing interventions on IR of falls is not always significant [44, 45]. Similarly, in our study, while no significant reduction in fall incidence was observed in the INT group compared to the CTR group, the tailored exercise program led to a positive trend for the reduction of the number of falls and a significant decrease in the number of PEF after adjusting for baseline fall history. These findings indicate the potential value of individualized interventions, particularly for enhancing functional outcomes, despite the lack of a significant impact on fall incidence.

Despite the absence of statistically significant changes in the number of falls and PEF within the INT group between baseline and 1-year follow-up, the statistically significant increase observed in the CTR group underscores the importance of interventions aimed at mitigating potential risks. Notably, IR of falls in the INT group exhibited a 12% increase, while the proportion of PEF decreased by 31%. Furthermore, the INT group demonstrated a 7.5% higher IR of falls compared to the CTR group after the study including the 1-year follow-up. However, after adjusting for baseline values, the INT group showed a 10.3% lower IR of falls than the CTR group at the end of the 1-year follow-up. These counterintuitive results may be associated with different fall assessment methods, retrospective reports of falls at baseline, and prospective use of fall diaries after baseline. Only the latter was based on daily documentation of falls that included specific details about the type of fall, such as falls without and with injury, and was further monitored through phone calls [46–48].

### Influence of baseline fall history

Fall history has been identified as a significant risk factor for falls [49, 50]. Despite the absence of a statistically significant difference in the number of falls between the groups, the high baseline fall incidence in the INT group (IR = 0.75, which exceeds the approximate maximum of 0.4 reported in other studies [1, 2, 51]) and the significantly lower number of PEF in the CTR group at baseline (see Tables 1 and 3) may have distorted the overall results. In contrast to the baseline, the incidence rate ratio (IRR) of falls and the risk ratio (RR) of PEF, both adjusted for baseline values, decreased by 45% and 61%, respectively. This finding broadly aligns with the outcomes of earlier studies that also demonstrated improvements, albeit without achieving statistical significance between groups [45, 52]. Specifically, one study demonstrated an approximate 35% reduction in the RR of PEF following a multifactorial intervention [53], while another exhibited a smaller reduction of around 3% [54]. The variation in these results [53, 55] may be due to differences in group characteristics and study design. The findings of the present study are more closely aligned with the study reporting a larger reduction [53], as both studies observed a higher RR of PEF in the INT group at baseline compared to the CTR group, which reversed at follow-up. Moreover, these enhancements appear to exceed those documented in preceding systematic reviews [16, 56]. However, the findings of our study suggest that the intervention is beneficial for general falls, but not for injurious falls. It should be noted that a pre-post analysis is not possible due to the absence of a distinction between falls without and with injury at baseline assessments. Injurious falls encompass a broad spectrum of outcomes, ranging from medical care to fractures and hospitalizations, reflecting substantial variability in their application in previous studies (see reviews by El-Khoury et al. and Schwenk et al. [57, 58]). Given that participants who experienced injurious falls continued with the intervention without dropping out, it can be assumed that the injurious falls in our study were likely minor or involved low-severity injuries.

### Hunova robot as a tailored intervention and diagnostic tool

The hunova robot used in the technology-supported phase provides standardized, task-specific balance challenges with real-time feedback and embedded assessment. Prior studies have reported good concurrent validity of hunova-derived SI with established performance measures and fall-risk classifications in older adults and clinical populations [32, 59, 60]. Our findings extend this evidence by demonstrating clinically meaningful improvements in the SI after the supervised phase and sustained advantages for the INT group after the HBEP and at follow-up. The small SI gains observed in controls likely reflect familiarization effects from repeated exposure to robotic assessments, which has been noted with balance platforms or sensor-based tests more broadly (see general test-retest learning effects discussed in studies measuring balance [61–64]).

Robotic-assisted sessions may enhance neuromuscular coordination and proprioceptive integration via high-repetition, error-augmented practice with immediate feedback, while the HBEP supports strength and functional mobility needed for transfer to daily activities. The sustained SI differences after HBEP suggest that a sequential hybrid model can promote retention, consistent with trials showing that partially supervised or hybrid programs improve physical performance in at-risk older adults [15, 16, 23, 24].

Despite risk reduction, we did not observe a statistically significant difference in fall incidence. This pattern is consistent with the multifactorial determinants of falls (e.g., medications, vision, environmental hazards, cognitive factors) emphasized in global guidelines [18] and with meta-analytic findings that effects on fall rate may be attenuated when programs are shorter, less frequent, or when co-interventions are limited [9, 12, 16]. It also underscores the importance of comprehensive, multi-component strategies for those at the highest risk [11, 12, 18].

Future interventions may need to incorporate a more holistic approach, addressing both intrinsic (e.g., balance, strength) and extrinsic (e.g., home environment, footwear) factors to further reduce fall incidence.

TUG is a standardized test used to assess mobility and is considered one of the gold standard methods for identifying older adults at high risk of falls [18]. TUG results in our study showed minimal changes in the INT group, with an average reduction of only 0.4 s, which is well below the threshold for clinically meaningful changes (typically 0.8 to 2.1 s; [65, 66]). The baseline TUG results, averaging around 8 s, may reflect a low baseline fall risk and a ceiling effect, as this is faster than the established cut-offs (13.5 to 15 s) used to identify fall risk in older adults [18, 55]. These findings suggest that TUG may be less sensitive for detecting fall risk changes in relatively healthy older adults, in line with previous research [67].

### Potential for implementing technology-supported and home-based exercise programs in older adults

Participants rated both TSEP and the HBEP as acceptable, with comparable effort and satisfaction. Interestingly, perceived temporal demand was higher for HBEP despite eliminating travel, suggesting that the structure and flow of TSEP (pre-set session goals, real-time guidance, automated progression) may have increased perceived efficiency. This is consistent with usability and implementation frameworks in which clear feedback and predictable session structure reduce cognitive load and support adherence [22]. Moreover, higher novelty scores for TSEP on the UEQ [36] likely contributed to motivation and engagement; factors repeatedly associated with better adherence in home- and group-based fall-prevention programs [15, 16, 23, 24].

From an implementation perspective, these findings resonate with the World Guidelines for Falls Prevention, which highlight the value of interventions that are acceptable, feasible, and scalable while delivering an adequate balance-specific dose [18]. Technology-supported delivery can help address known barriers to participation (transportation, supervision needs, tailoring) noted in exercise-based falls prevention [16, 17, 19, 20], while the sequential hybrid design may balance efficiency (center-based standardization) with sustainability (home-based maintenance). Future work should report acceptability and feasibility with standardized metrics (e.g., adherence, retention, adverse events) to facilitate comparison and scale-up [22].

The adherence rates for both programs were relatively high (84% for TSEP and 88% for HBEP), exceeding adherence rates reported in other fall prevention studies (76%; [44]). However, the dropout rate during TSEP was higher (26%) compared to HBEP (11%), with most participants citing lack of motivation for dropping out of TSEP, while health issues were the primary reason for dropouts during HBEP. These findings highlight the importance of balancing novelty and motivation with the need for program flexibility and accessibility. Although a review study reported dropout rates of 10% during the 12-month follow-up phase and 20% during the intervention phase for older adults [68], another review examining dropout rates in training programs using exergames for older adults demonstrated a wider range, from 0 to 40% [69].

The high acceptability and adherence rates observed in both the robot-assisted and home-based components suggest that this two-phase intervention could be feasible for wider implementation in community settings. However, its applicability in less controlled environments, such as rural or low-resource areas, needs further exploration. The requirement of a specialized device (hunova robot) limits the scalability of the program to locations with access to such technology, and the cost of robotic equipment may be prohibitive for widespread use. On the other hand, the home-based phase, which does not require specialized equipment, has significant potential for scalability and could be adapted into community-based fall prevention programs or integrated into tele-rehabilitation initiatives. A recent study found that technology-assisted exergame training, conducted at home using a mat, significantly reduced fall incidence and fear of falling in healthy older adults [70]. Policymakers and health organizations should consider ways to subsidize or provide access to technology-assisted training for older adults, particularly in higher-risk populations, to maximize the benefits of such tailored interventions.

### Limitations

The ceiling effect observed in the TUG test and gait speed in both the INT and the CTR group indicates that our post measurements may not have been sensitive enough to detect meaningful changes in fall risk in our study population. Therefore, using the TUG with a recommended threshold of 15 s or gait speed with a threshold of 0.8 m/s, as recommended by the current World Fall Guidelines [18], or a comprehensive fall risk assessment focusing on physiological functions, such as the physiological profile approach [71], may be more appropriate as inclusion criteria than the one applied in this study, such as SI.

One limitation of this study is its focus on a relatively healthy population of older adults, which limits the generalizability of the results to higher-risk populations who could benefit more from fall prevention interventions. The population utilized for the estimation of the sample size exhibited a mean age of 76 years, with 77% identifying as female. Additionally, the sample included a modest proportion of individuals recently discharged from the hospital (4%) [72]. In contrast, the participants in our study were relatively younger, had a lower proportion of females, and were generally fitter, which may have contributed to the smaller intervention effects observed. It is possible that a much larger cohort of such fit and healthy older adults may be necessary to detect significant differences in fall incidence.

It is crucial to acknowledge the distinction between the methodologies employed in the collection of fall data at baseline (retrospective) and after baseline (prospective), including potential variations in the fall definition due to differences in questioning versus documentation, as well as differences in the type of falls recorded (e.g., falls in general at baseline vs. specific types of falls after baseline). Given the possibility of underreporting in retrospective data [73, 74], the fall incidence observed at follow-up may reflect the enhanced accuracy of prospective data collection, potentially attenuating the intervention effects. Thus, future studies should standardize methodologies for data collection and the operational definition of falls to reduce potential inconsistencies. Additionally, studies should explore whether combining this exercise program with other fall prevention strategies, such as environmental modifications or medication use, could result in a more significant reduction in fall incidence. Given the promising results for fall risk reduction, long-term studies are needed to assess whether the improvements in balance and strength translate into sustained reductions in falls over time. Furthermore, cost-effectiveness analyses should be conducted to determine whether the initial investment in robotic technology is offset by long-term healthcare savings, particularly in preventing falls that lead to injury and hospitalization.

## Conclusion

The results of our investigation indicate that the two-phase exercise program, which combines robot-assisted and home-based training, has the potential to reduce the risk of falls in older adults, as evidenced by improvements in the SI. Nevertheless, the absence of statistically significant training effects on fall incidence between the two groups indicates the potential necessity for further intervention refinement, particularly for relatively fit and healthy older adults. Both phases of the program were generally well accepted and feasible, with high adherence and motivation, indicating promise for wider implementation. It is recommended that future research focus on adapting the exercise intervention to enhance its efficacy in both relatively healthy and older adults at an elevated risk of falls. This should be done in a manner that maintains consistency and validity in the documentation of falls and fall risk assessment methods.

## Supplementary Information

Supplementary Material 1.

Supplementary Material 2.

## Data Availability

The datasets analyzed during the current study are available from the corresponding author on reasonable request.
