# Supplementary material for: Impact of a two-phase robotic-assisted and home-based training program on falls and fall risk in older adults: a multicenter randomized controlled trial
Source: Trials. 2025 Dec 20;27:80. doi: 10.1186/s13063-025-09380-x (PMC12831356; doi:10.1186/s13063-025-09380-x)
Supplement: Supplementary file 1 — Supplementary Material 1. [file 13063_2025_9380_MOESM1_ESM.docx]

**Appendix: Baseline characteristics of the study participants at T_0_**

|  |  | INT (*n*=147) | CTR (*n*=145) | *p* |
| --- | --- | --- | --- | --- |
| Gender, female (*n* (%)) | | 75 (51.02) | 79 (54.48) | .554 |
| Age (yrs) | | 72 (6.03) | 73 (5.19) | .225 |
| Body height (cm)^*^ | | 172.4 (9.33) | 172.3 (9.84) | .916 |
| Body mass (kg)^†^ | | 81.9 (15.71) | 81.5 (15.17) | .988 |
| BMI (kg/m²)^†^ | | 27.5 (4.29) | 27.4 (3.99) | .823 |
| SI value (%) | | 66.35 (16.98) | 63.80 (16.80) | .199 |
| Gait speed (m/s) | | 1.20 (0.20) | 1.25 (0.18) | .052 |
| TUG time (s)^‡^ | | 8.65 (2.22) | 8.28 (1.66) | .273 |
| Falls in the past year (*n* (%)) | |  |  | .442 |
|  | No falls | 92 (62.59) | 97 (66.90) |  |
|  | Fall(s) | 55 (37.41) | 48 (33.10) |  |
| Overall health status (EQ-5D-5L)^±^ | |  |  |  |
|  | Mobility | 1.33 (0.71) | 1.19 (0.52) | .113 |
|  | Self-care | 1.08 (0.41) | 1.06 (0.38) | .801 |
|  | Usual activities | 1.20 (0.54) | 1.10 (0.45) | *.008* |
|  | Pain / discomfort | 1.66 (0.78) | 1.56 (0.72) | .337 |
|  | Anxiety / depression | 1.22 (0.55) | 1.09 (0.32) | *.046* |
|  | Health status | 78.64 (15.52) | 81.10 (15.14) | .100 |
| Physical activity (German PAQ-50^+^)^§^ | | |  |  |
|  | Activity (MET-hours/week) | 139.97 (72.93) | 143.25 (88.43) | .845 |
|  | Energy (kcal/week) | 10928.23 (6118.61) | 11329.08 (7253.07) | .910 |

Data are given as mean (*SD*: Standard deviation) unless stated otherwise. ^*^*n*: CTR = 144; ^†^*n*: INT = 146, CTR: 144; ^‡^*n*: INT = 144, CTR = 140; ^±^*n*: INT = 143, CTR = 140; §*n*: INT = 66, CTR = 85.

*Note.* INT: Intervention group; CTR: Control group; BMI: Body Mass Index; SI: Silver Index; T_0_: Baseline measurement; TUG: Timed-up-and-go test.
